# Editorial Notes

**Published:** 1934

**Authors:** 


### Editorial Notes

**Winford Orthopædic Hospital.**

As the result of the voluntary winding-up of Bristol Housing Ltd., formerly known as Bristol Garden Suburb Ltd., an additional ward is to be built and equipped at the Winford Orthopædic Hospital.

The original company, known as the Bristol Garden Suburb Ltd., was formed in 1909, for the purpose of developing an estate of 26½ acres at Shirehampton on garden suburb lines.

Now that the Government and the municipal authorities have taken the matter in hand so energetically, and so many private builders being forthcoming, the reason for the existence of the company no longer obtains, and it has been decided to wind up its affairs, repaying in full the capital invested with interest to date.

During the twenty-five years of its existence the company has accumulated a modest reserve, and this, together with the increase in the value of the company's ground-rents brought about by the cheapness of money, is sufficient to enable the directors (with the consent of the shareholders, most readily and cheerfully given) to offer to build and equip an additional ward at the Winford Orthopædic Hospital, which is so urgently required.

The actual sum offered is £10,000, and it is estimated that this will cover the cost of erecting and equipping a new ward with 56 beds, thus making a total of 112 beds available. Such an extension ought to enable the authorities to admit children in the early stages of rheumatic heart disease without the delay which the present long waiting list involves. The chief object of maintaining a heart hospital school at Winford is the early admission of children suffering from rheumatic fever whilst the damage to the heart may be hoped not to be permanent. The more doubtful the heart disease the more useful will be the treatment at Winford, since it is almost certain that rheumatic fever affects every heart in a greater or lesser degree.

**Bicentenary of the Royal Infirmary.**

THE Committee of the Royal Infirmary announce that the Bicentenary of the Institution will be celebrated in 1937. The reason for choosing this date is that the Infirmary was formally opened for the reception of patients on 13th December, 1737. Although the date of the foundation has been given as 1735, there exists a " Vellum Book " in which the roll of subscribers is inscribed, and no subscription is given a date prior to 1736. This is the " Vellum Book " mentioned by Munro Smith in his history of the Bristol Royal Infirmary as having been lost. Mr. Ellis Smith has, however, been fortunate enough to find it amongst the minute books and archives.

In 1837 the Committee discussed at several meetings how they should celebrate the Centenary. Apparently nothing was done to mark the occasion, because a dispute arose between the Committee and Medical Staff which occupied their minds. But in the " State " or Annual Report for 1837

one subscriber is shown to have remembered the occasion. On December 16th, 1837, Mrs. Martha Daubeny, of Henbury, sent a "Centenary donation" of £100.

That is how the Centenary of the Bristol Royal Infirmary was kept. It is to be hoped that at the Bicentenary Mrs. Martha Daubeny's example will be borne in mind.

